# A Quasi-Experimental Study of a Movement and Preliteracy Program for 3- and 4-Year-Old Children

**DOI:** 10.3389/fped.2017.00094

**Published:** 2017-05-01

**Authors:** Chloe Bedard, Emily Bremer, Wenonah Campbell, John Cairney

**Affiliations:** ^1^INfant and Child Health (INCH) Lab, Department of Health Research Method, Evidence, and Impact, McMaster University, Hamilton, ON, Canada; ^2^INfant and Child Health (INCH) Lab, Department of Kinesiology, McMaster University, Hamilton, ON, Canada; ^3^INfant and Child Health (INCH) Lab, Department of Rehabilitation Sciences, McMaster University, Hamilton, ON, Canada; ^4^Faculty of Kinesiology and Physical Education, University of Toronto, Toronto, ON, Canada

**Keywords:** early childhood, fundamental movement skills, preliteracy skills, early intervention, child development

## Abstract

**Objective:**

Approximately 28% of children are not ready for kindergarten, 91% are inactive according to current guidelines, and 21% are overweight/obese. Early intervention to strengthen movement and preliteracy skills may help to curb the concerning rates of poor school readiness, inactivity, obesity, and subsequently positively impact health across the lifespan. The objective of this pilot study was to evaluate the effectiveness of a motor and preliteracy skill program for a community sample of 3- to 4-year-old children.

**Methods:**

A quasi-experimental study design was used. The program was run for 1 h/week for 10 weeks and consisted of movement skill instruction, free play, and an interactive reading circle with care-giver involvement throughout each session. Movement and preliteracy skills were assessed in all children pre- and post-intervention using the Peabody Developmental Motor Scales-2nd edition, the Preschool Word and Print Awareness tool, and the Phonological Awareness Literacy Screening tool.

**Results:**

Nineteen families (experimental group, *n* = 8; control group, *n* = 11) were recruited (mean age = 3 years, 8 m; 47% male). There was a significant effect of group on gross motor raw scores overall [*F*(1, 16) = 4.67, *p* < 0.05; $\omega_{\text{p}}^{2}=0.16$] and print-concept knowledge [*F*(1, 16) = 11.9, *p* < 0.05; $\omega_{\text{p}}^{2}=0.38$].

**Conclusion:**

This study was one of the first to examine the impact of a community-based movement skill and preliteracy program with care-giver involvement in preschool children. Future research should continue to explore the effects of the program with larger and more diverse samples on multiple health and developmental outcomes.

**Clinical Trial Registration:**

Play and Preliteracy among Young Children (PLAY) NCT02432443.

## Introduction

There is mounting evidence linking health status and behaviors in childhood to future health problems in adolescence, adulthood, and later adulthood (1, 2). For example, children who are overweight or obese are more likely to be overweight as adults and suffer from cardiovascular disease and poor mental health (3, 4). In response to this evidence, a great deal of research has focused on optimizing childhood development in order to improve health outcomes across the lifespan. However, in a Canadian population of children, 28% are not ready for school (5), 91% are insufficiently active according to current guidelines (6), and 21% are considered overweight or obese (7). Given the high prevalence of inactivity, overweight/obesity, and poor school readiness, we can conclude that significant improvements need to be made in order to enhance the health and development of children over time. Extant research has targeted children who have developmental delays or who are of school age, leaving a critical gap in the literature for younger, preschool-aged children with typical development (8). There is value in attending to this large part of the population as the potential impact of intervening in this population is large.

Underlying the rise in physical inactivity, poor school readiness, and obesity rates is hypothesized to be poorly developed motor and cognitive skills, particularly gross motor and preliteracy skills. This hypothesis emerges from the literature relating motor proficiency with physical, psychosocial, and cognitive development as well as evidence linking the development of preliteracy skills with psychosocial and cognitive domains (9–11). With respect to motor development, it has been shown that the attainment of fundamental movement skills (FMS) such as throwing and catching allow children to learn more complex movements that facilitate independent participation in activities of daily living, athletic pursuits, and physical activity (PA) (12). Engagement in these types of activities improves physical development through increases in PA levels, physical fitness, and healthy body composition (8, 13). In addition, increasing evidence from neuroscience research suggests movement skills in childhood are inextricably linked to other core aspects of development including social, cognitive, and psychological domains (14–17). With respect to preliteracy skills, studies have demonstrated relationships between early language and literacy development and later socioemotional regulation (18), social behavior (9), and academic self-perceptions (10).

There is reasonable theoretical, empirical, and practical evidence to promote a component-based program that teaches movement and preliteracy skills. From an embodied cognition perspective, motor and cognitive developments are intrinsically linked as cognitive processes, in particular language, emerge as individuals interact with their physical and social environment (19). As children develop their movement skills and interact with their surroundings, they have more diverse opportunities to learn about their environment and develop their language and other cognitive abilities (20, 21). Early language and preliteracy skills are intimately linked through their role in communication (22) thus, by extension we may apply the embodied cognition perspective to a paired movement skill and preliteracy skill-learning paradigm, in which we may expect enhanced learning. Empirical evidence supports this relationship between movement and preliteracy skills. Callcott et al. (23) demonstrated the synergistic benefits of a blended learning program on both movement and preliteracy outcomes. When preliteracy skill-building activities were used as a medium to teach movement skills, children showed greater improvements compared to a group of children participating in a movement-only program (23). From a practical perspective, including both reading activities and movement skill-building activities in a single program will attend to both parental and kindergarten curricular interests. Parents and educators of young children are concerned with achievement of both motor milestones and school readiness indicators; therefore, by targeting both domains in a single program, we will appeal to various child care providers. Finally, skill-building activities for both movement and preliteracy development are fun and enjoyable for children and their families (24, 25). Learning how to jump and throw and sharing a book with their caregivers are intrinsically appealing to children, which is an important predictor of participation and engagement (26).

It is critical to consider the role of the parents in these programs. Parents act as a child’s primary instructor in the early years when children are learning new skills and with effective strategies they can support their development in a number of domains (27–29). The involvement of parents in skill-building programs allows for skill learning to continue outside of the program setting. Systematic reviews have consistently supported the inclusion of parents throughout programs and the dissemination of parent materials to facilitate knowledge translation into the home in order to achieve sustained improvement in movement skill proficiency (27, 30).

In light of all of the aforementioned considerations, we designed an evidence-based intervention targeting movement and preliteracy skills in children 3–4 years of age. The objective of this study is to evaluate the effectiveness of the program.

## Methods

### Design

A quasi-experimental study design was used to evaluate the program. All children were assessed twice approximately 10–12 weeks apart: children in the experimental group were assessed pre- and post-program; children in the control group were assessed at baseline and again following 10 weeks. Families had the option of participating in the experimental group or control group, depending on their preference for the start date (Summer or Fall). Families in the control group were able to participate in the program after their second appointment was completed.

### Participants

A convenience sample of families was recruited through advertisements at Ontario Early Years Centres, Boys and Girls Clubs of Hamilton, various licensed daycares, and the Hamilton Public Libraries from May to July 2015. Children were eligible to participate if they were between the ages of 3 years, 0 months and 4 years, 11 months at baseline and must not have been diagnosed with any developmental delay or other health condition that would prohibit safe participation in the program.

### Intervention

The program took place at the Boys and Girls Club of Hamilton and was led by two graduate students (Emily Bremer and Chloe Bedard under Dr. Cairney’s supervision). The program ran for 60 min once per week for 10 consecutive weeks and consisted of three components: direct FMS instruction, unstructured exploratory free play, and a dialogic storybook reading activity. The first segment of the program focused each week on a specific movement skill through single-step skill acquisition strategies (i.e., introducing new skills one-by-one). The free-play segment of the program allowed children the opportunity to self-direct their own activities: children had access to various play items including both gross motor and fine motor equipment (e.g., playground balls and puzzle pieces). The curriculum and teaching strategies used for the first two segments of our intervention have been successfully implemented in previous research to improve the movement skills of preschool-aged children with autism (31, 32); these strategies have been adapted for use with our general population of children. The final segment of our program was a dialogic shared book reading circle with all the children and their parents. Each week used one book to develop one to two preliteracy skills. Specific strategies and books were selected from several evidence-based curricula (25, 33, 34). There was active involvement of at least one parent in the direct instruction and reading components.

### Outcome Measures

#### Demographic and Engagement Survey

The demographic questionnaire was completed at baseline and included questions about the parent and the child on age, gender, ethnicity, parental education and occupation, and household income. A parent engagement questionnaire was administered at both assessments and was developed to assess the use and the frequency of use, of specific activities that were employed in our program to determine if parents use these activities at home.

#### Preliteracy Skills

Children were administered the Preschool Word and Print Awareness tool (PWPA) test and the Phonological Awareness Literacy Screening: preschool (PALS-PK)-uppercase alphabet recognition task at both assessments to measure print-concept knowledge and alphabet knowledge (35, 36), respectively. The PWPA tests children on their print-concept knowledge, such as print directionality and print function, using 14 items administered in an interactive storybook reading format (35). Raw scores are then transformed into standardized scores with a mean of 100 and SD of 15. The PWPA has strong validity, and the reliability is 0.74 in a sample of children aged 3–5 years (37). The PALS-PK uppercase alphabet recognition task involves children naming each of the 26 letters of the alphabet as they are presented in a random order. The inter-rater reliability coefficient of this task is 0.99 (36). These measurements together took approximately 15 min to complete and were administered by a trained graduate student.

#### Motor Skills

Children were administered the gross motor subtests of the Peabody Developmental Motor Scales-2nd edition (PDMS-2) (38) at both assessments. The PDMS-2 is a standardized instrument designed to measure the progress of development of gross and fine motor skills in children from birth to age 6. The three gross motor subtests—stationary, locomotion, and object manipulation—were administered by two trained graduate students. The sum of the raw scores of each of the three subtests was used as the dependent variable in the primary analysis. The assessment required approximately 30–45 min to conduct (39). The PDMS-2 has good validity and sensitivity to change as assessed previously in 4-year-old typically developing children and the inter-rater reliability is 0.89 (39, 40).

#### Attendance and Home Practice

Attendance at the program sessions and the frequency of weekly home practice was measured using attendance checklists and parent-reported questionnaires.

### Procedure

**Figure 1:**
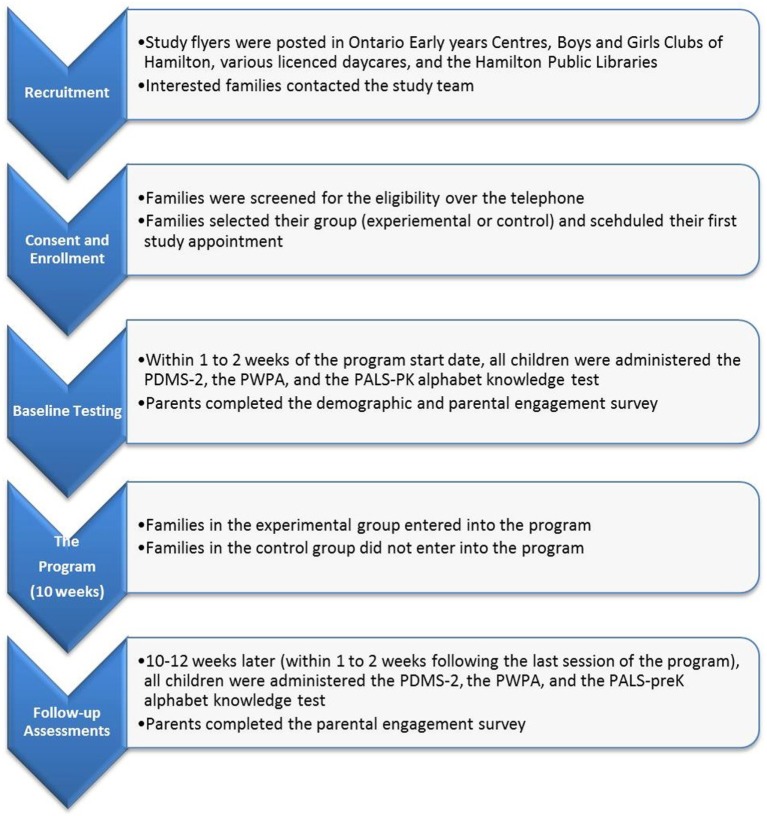
**Flow diagram for study procedures**.

The study received ethical approval from the Hamilton Integrated Research Ethics Board. All study appointments took place at the INfant and Child Health (INCH) Lab at McMaster University. After eligibility was confirmed over the telephone, parents were asked to select a time for their first study appointment. Informed written consent was obtained at the first study visit. At each appointment children were administered the PWPA, PALS-PK—uppercase alphabet recognition task to assess preliteracy skills, and the PDMS-2 to assess movement skills. Parents were asked to complete the demographic (at the first assessment only) and the engagement questionnaire. The duration of each appointment was approximately 1–1.5 h. After the first appointment, families were asked to select their group (experimental/summer start or control/fall start). Families in the experimental group began the program within 1–2 weeks of their baseline appointment. After the experimental group completed the program, all families were asked to come in for their second assessment in which they were administered the same tests. See Figure 1 for the flow diagram depicting the study procedures.

### Statistical Analyses

Basic descriptive statistics of the demographic characteristics of the sample and attendance and at-home practice rates were computed. Between-group differences in baseline characteristics were compared using independent *t*-tests for continuous variables and chi square (Fischer’s exact test) for categorical variables. The primary analyses were three group by time analyses of covariance (ANCOVAs) to assess changes in the children’s gross motor skills (raw scores), print-concept knowledge (standardized scores), and alphabet knowledge between the experimental and control groups at time 2 with time 1 scores entered as a covariate. *Post hoc* ANCOVAs were used to examine differences across time in each gross motor subtest between groups. Secondary analyses included two group by time ANCOVAs examining differences in changes in parental engagement in both movement and preliteracy skills. The partial omega squared will be used as a measure of effect size as it calculates the variance accounted for by group assignment controlling for the time 1 scores. This effect size is appropriate for analyses with more than one independent variable (i.e., an ANCOVA) (41). Effect sizes are interpreted as small for values over 0.01, medium for values over 0.06, and large for value over 0.14 (42). All analyses were conducted on a per-protocol basis. A two-tailed alpha value of 0.05 was used to determine statistical significance.

## Results

### Descriptives

**Figure 2:**
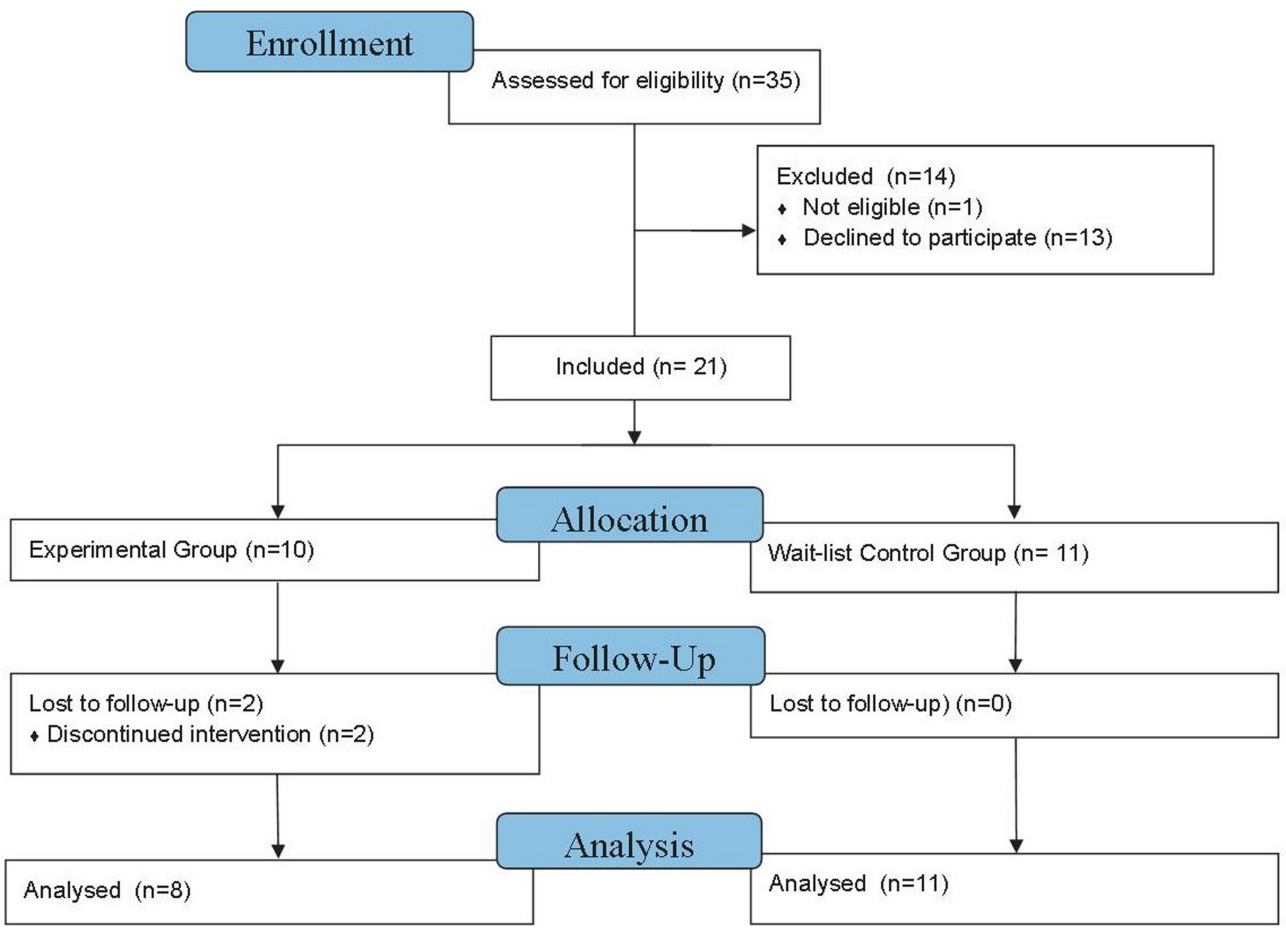
**Flow diagram to indicate the included and excluded participants**.

Twenty-one families were eligible and consented to participate in the first study appointment (see Figure 2). Of these 21 families, 19 completed the second study visit: 2 families discontinued the program and were lost to follow-up; 1 family withdrew from the intervention due to time constraints, however, attended the second study appointment. The final study sample included 19 children (10 boys) ranging from 3 years, 0 months to 4 years 11 months (mean = 3 years, 8 months, SD = 7.30 months).

Table 1 describes the demographic characteristics of the sample by group. There were no significant differences between the two groups for any demographic characteristic, with the exception of parental education (see Table 1). Baseline scores on raw and age-standardized gross motor skills, print-concept knowledge, and alphabet knowledge were not significantly different between groups (see Table 2). The median attendance level was 8 out of 10 program sessions (IQR: 7–9 sessions). The average rate of home practice was 45% (SD = 17%) and 52% (SD = 18%) for the movement and preliteracy activities, respectively.

**Table 1:** **Sample demographic characteristics of the experimental and control groups**.

Variable	Experimental group (*n* = 8)	Control group (*n* = 11)	*p*-Value
**Child’s mean age in months (SD)**			
	41.4 (6.99)	45.6 (7.30)	0.22
**Child’s gender %**			
Male	51	55	>0.99
**Child’s ethnicity %**			
Filipino	12	0	0.77
Black	0	9	
South Asian	0	9	
Chinese	12	0	
Mixed ethnicity	13	9	
White	63	73	
**Parent age (years)**			
	37.5	33.0	0.09
**Parent education %**			
College/technical training	0	45.5	0.04
University degree	100	54.5	
**Parent income %**			
Less than $50,000	12	36	0.27
Greater than $50,000	87	64	

*n, sample size*.

**Table 2:** **Baseline scores of the experimental and control groups**.

Variable	Experimental group (*n* = 8)	Control group (*n* = 11)	*p*-Value
	Mean (SD)	Mean (SD)	
**Gross motor skill (raw)**			
Stationary (SD)	46.0 (6.63)	47.5 (5.47)	0.61
Locomotion (SD)	140.0 (22.42)	150.7 (12.20)	0.20
Object manipulation (SD)	28.4 (7.13)	33.8 (5.56)	0.08
Total	214.4 (35.38)	232.0 (19.57)	0.18
**Preliteracy skill**			
Print-concept awareness (SD)	87.5 (16.49)	93.9 (19.72)	0.47
Alphabet knowledge (SD)	10.6 (0.45)	11.3 (8.89)	0.89

*n, sample size; SD, standard deviation*.

### Intervention Effects

**Figure 3:**
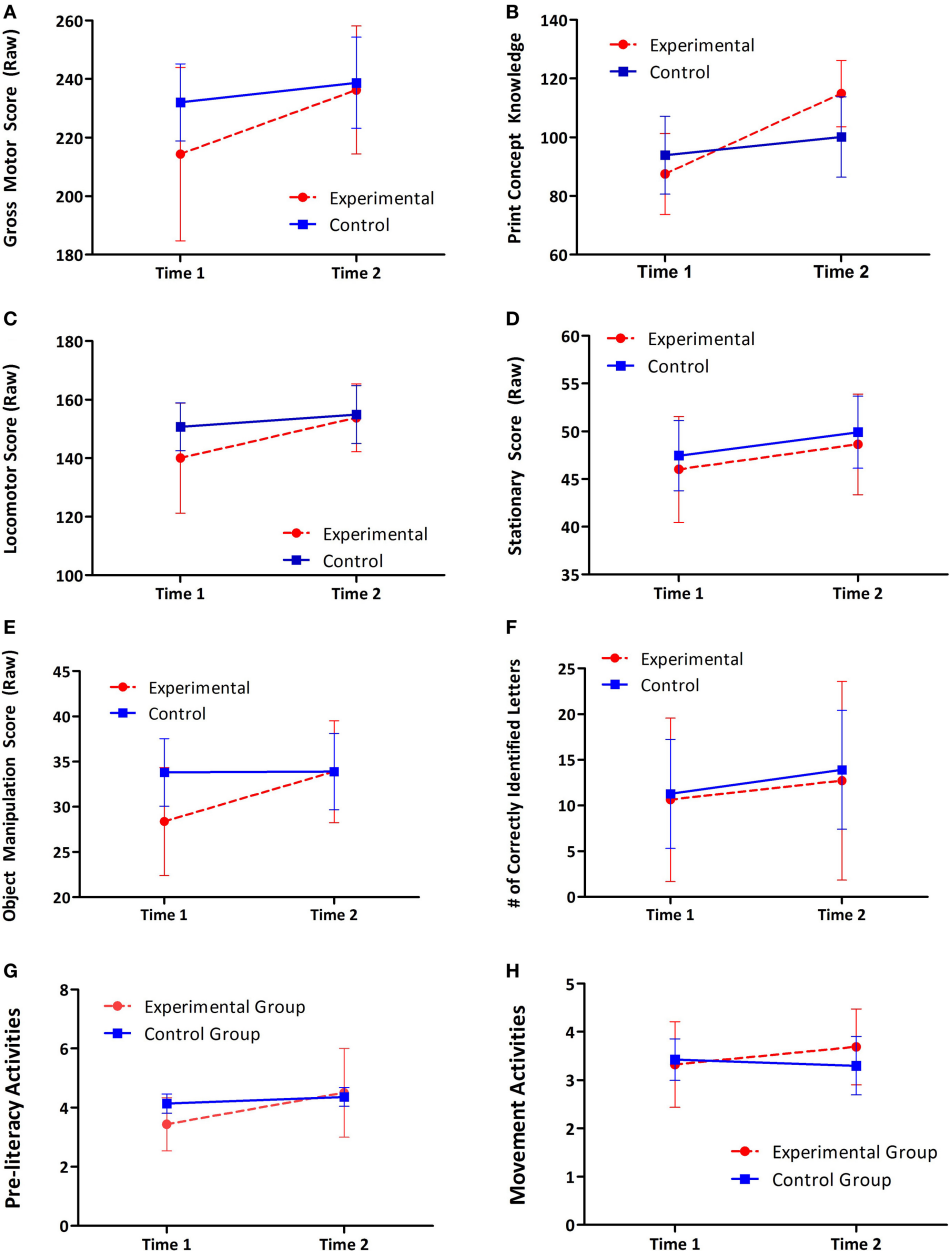
**Mean scores at time 1 and time 2 for both groups; error bars represent the 95% confidence intervals**. **(A)** Gross motor skills; **(B)** print-concept knowledge; **(C)** locomotor skills; **(D)** stationary skills; **(E)** object manipulation skills; **(F)** alphabet knowledge; **(G)** parental preliteracy engagement scores; and **(H)** parental movement engagement scores.

**Figure 4:**
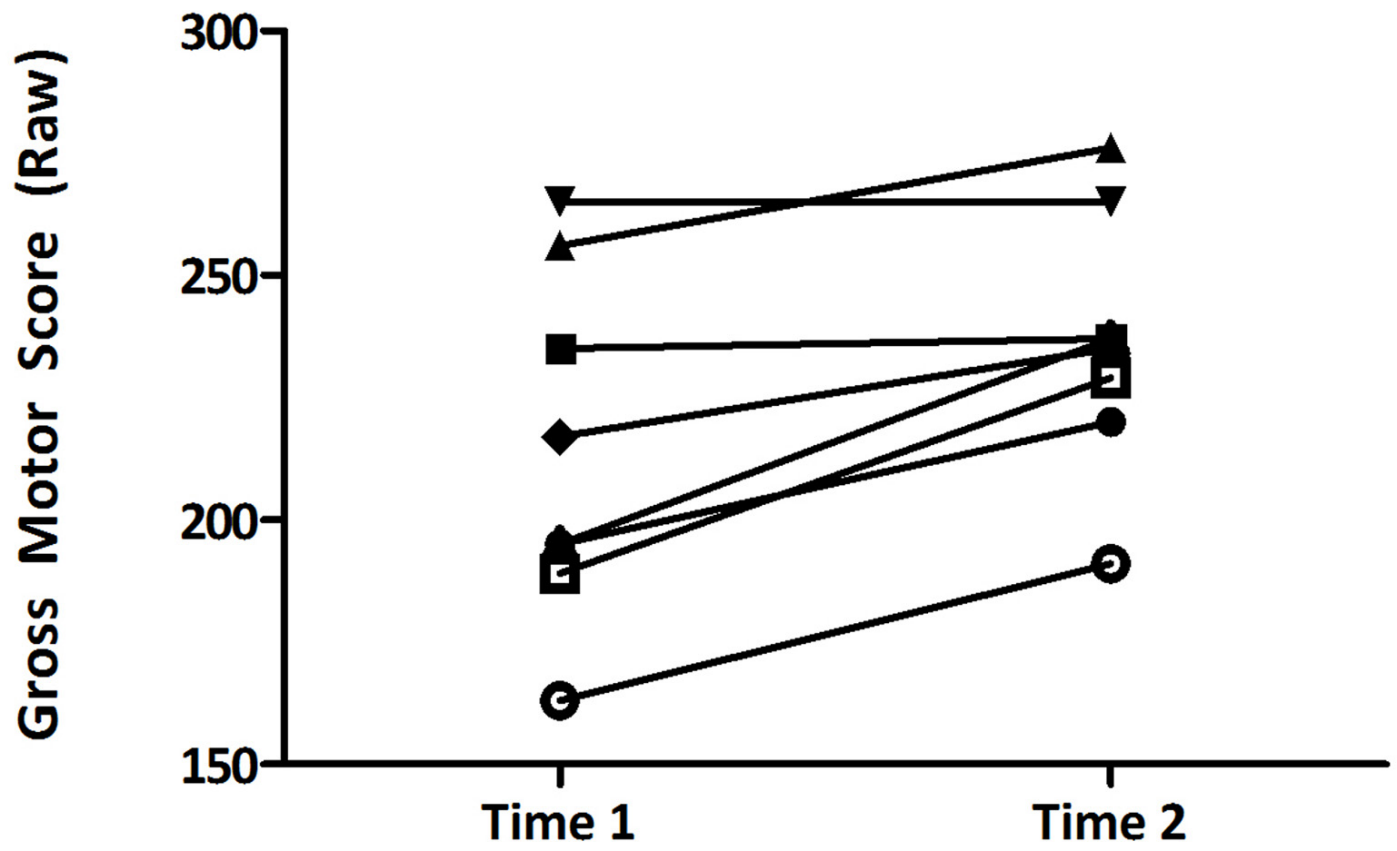
**Individual change over time in gross motor skills in the experimental group**.

Mean scores and SDs of the primary outcomes (movement and preliteracy skills) at each time point by group are presented in Table 3. Overall, both group improved their movement and preliteracy skills over the 10 weeks (see Table 3). Parents showed small increases in their levels of engagement, with the exception of parents in the control group and their engagement in motor activities (see Table 3). Results of the ANCOVA on the primary outcomes are presented in Table 4. There was a significant group effect on gross motor raw scores measured at time 2, after controlling for time 1 scores [*F*(1, 16) = 4.67, *p* < 0.05; $\omega_{\text{p}}^{2}=0.16$]. There was also a significant effect of group on print-concept knowledge [*F*(1, 16) = 11.9, *p* < 0.05; $\omega_{\text{p}}^{2}=0.38$]; however, improvements in uppercase letter recognition did not differ significantly between groups [*F*(1, 15) = 0.048, *p* = 0.83; $\omega_{\text{p}}^{2}<0.001$]. *Post hoc* analyses reveal that there was a significant difference in the improvement of object manipulation skills in the experimental group, compared to the control group; improvements in scores on the stationary and locomotor domains were not significantly different between groups (see Table 5). The results of the secondary analyses indicate non-significant differences between the experimental and control group on their level of engagement in both movement and preliteracy activities (see Table 6). Figures 3A–H show the change over time in the experimental and control group for each primary and secondary outcomes. Figure 4 illustrates the change over time in the gross motor scores of the individual participants in the experimental group. There were no reported adverse effects of the intervention.

**Table 3:** **Scores for each assessment in the experimental and control groups for gross motor and preliteracy skills, and parent engagement scores**.

Variable	Experimental (*n* = 8)		Control (*n* = 11)	
	Time 1	Time 2	Time 1	Time 2
	Mean (SD)	Mean (SD)	Mean (SD)	Mean (SD)
Gross motor skill—raw score	214.38 (35.37)	236.25 (26.11)	232.00 (19.57)	238.72 (23.20)
Print awareness—standardized score	87.50 (16.49)	114.90 (13.49)	93.91 (19.72)	100.10 (20.38)
Uppercase letter recognition	10.63 (10.69)	12.71 (11.74)	11.27 (8.90)	13.91 (9.68)
Parent engagement—motor	3.33 (1.06)	3.70 (0.94)	3.43 (0.64)	3.30 (0.91)
Parent engagement—preliteracy	3.43 (1.07)	4.50 (1.80)	4.14 (1.09)	4.36 (1.06)

*n, sample size*.

**Table 4:** **Analysis of covariance results for primary outcomes of motor and preliteracy skills**.

Source	*F*-value	*p*-Value	$\omega_{\text{p}}^{2}$
**Total gross motor score**			
Group	4.67	<0.05	0.16
Time 1 scores	66.74	<0.05	
**Print-concept knowledge scores**			
Group	12.66	<0.05	0.38
Time 1 scores	23.94	<0.05	
**Uppercase letter recognition**			
Group	0.048	0.83	<0.001
Time 1 scores	148.42	<0.05	

*The dependent variables are the time 2 scores (df numerator = 1, denominator = 16)*.

*df, degrees of freedom;* $\omega_{\text{p}}^{2}$*, partial omega squared*.

**Table 5:** ***Post hoc* analysis of covariance results**.

Source	*F*-value	*p*-Value	$\omega_{\text{p}}^{2}$
**Stationary**			
Group	0.00	0.99	<0.001
Time 1 scores	58.74	<0.05	
**Locomotion**			
Group	2.40	0.14	0.07
Time 1 scores	35.00	<0.05	
**Object manipulation**			
Group	6.13	<0.05	0.21
Time 1 scores	36.38	<0.05	

*The dependent variables are the time 2 scores (df numerator = 1, denominator = 16)*.

*df, degrees of freedom;* $\omega_{\text{p}}^{2}$*, partial omega squared*.

**Table 6:** **Analysis of covariance results for secondary outcomes**.

Source	*F*-value	*p*-Value	$\omega_{\text{p}}^{2}$
**Parental engagement—motor**			
Group	2.32	0.15	0.06
Time 1 scores	16.95	<0.01	
**Parental engagement—preliteracy**			
Group	3.45	0.08	0.11
Time 1 scores	23.69	<0.01	

*The dependent variables are the time 2 scores (df numerator = 1, denominator = 16)*.

*df, degrees of freedom;* $\omega_{\text{p}}^{2}$*, partial omega squared*.

## Discussion

The aim of this study was to investigate the impact of a program designed to improve preschool-aged children’s movement and preliteracy skills and the results show large significant effects between the experimental and control group in the improvement in both skill domains. This study is timely as evidence continues to accumulate supporting the critical importance of movement and preliteracy skills to several health and academic outcomes in preschool-aged children (43, 44). This alongside the growing number of children who are physically inactive (6), overweight or obese (7), and not ready for school (5) signals that targeted multicomponent interventions are necessary. An opportune time to intervene exists during the early years because children typically find enjoyment in learning new movement skills, such as jumping and throwing, and reading stories; thus, finding movement- and reading-based programs fun (24).

It is important to consider the results of this intervention within the context of the program structure. The positive improvements in gross motor skills suggest that programs consisting of direct movement skill instruction have the capacity to improve the movement skills of children with typical development. This aligns with the few intervention studies conducted in this age group showing positive gains in children participating in direct movement skill instruction (27). Furthermore, the significant gains in both gross motor and preliteracy skills despite the relatively low dose of the intervention (i.e., one time per week for 1 h) highlight the importance of both the combined learning environment and the parental component. As demonstrated by Callcott et al. (23), pairing motor and preliteracy learning in a single program has the capacity to produce synergistic gains in both domains. Although this synergistic hypothesis was not directly tested in this study, our results suggest that important gains in movement and preliteracy skills can be achieved in a dual program that capitalizes on this relationship. Additionally, parents were actively involved in the program and were provided with handouts outlining the weekly activities and encouraged to practice the activities at home. The rate of at-home practice for both the movement and reading activities ranged from 45 to 52%, indicating that activities were practiced on approximately half the days not spent in the program. The involvement of the parents was likely critical to expand their child’s learning beyond the weekly session. One recommendation from Veldman et al. (27) suggests that programs should run at least twice a week; however, our results suggest that running one session a week and involving parents actively throughout the program may confer similar skill gains to a higher-dose program.

The results of the *post hoc* analyses on our primary motor outcomes indicate that the program seems to selectively improve object manipulation skills, not stationary or locomotor skills. However, there was a medium effect size observed for locomotor skills suggesting that non-significant findings for this domain may have been a power issue. The non-significant findings with respect to stationary and locomotor skills suggest that the movement skill component of the program should be modified to enhance skill development in these domains. We also found selective improvements in print-concept knowledge and not in alphabet knowledge. This may be explained through the organization of the program: print concepts were introduced during the first half of the intervention and alphabet concepts were introduced in the latter half. Thus, children were exposed to print concepts for a longer duration over the intervention period, compared to alphabet concepts, lending to the observed results. Finally, we also found non-significant findings with respect to levels of parental engagement of motor and preliteracy activities at home, and this may explained in consideration of the parental component of the intervention and measurement issues. The effect size for changes in parental engagement in motor and preliteracy activities at home are moderate and large, respectively; therefore, the analyses may be limited in their power to detect a statistical difference even when these fairly substantial increases exist. Furthermore, the measurement of engagement was through a self-reported survey, which may not be sufficiently sensitive to detect significant differences. Finally, the survey items asked about only the frequency of engagement, not the quality of practice. Therefore, while we could not detect changes in frequency of practice of activities at home, it may be that parents engaged in infrequent, but high-quality practice at home.

There are limitations that should be noted in conjunction with the results of the study. First, participants were not randomly assigned to the intervention and control group, thus there are some imbalances in baseline characteristics. The imbalances in baseline motor and preliteracy skills were non-significant and were accounted for in the analyses; the effect of the intervention remained significant after controlling for these differences in time 1 scores. However, it remains possible that our results reflect greater opportunity for improvement in the experimental group given their lower baseline scores. Thus, future research should ensure that baseline movement and preliteracy scores are equivalent in both groups. The imbalance in parental education levels may have impacted the results through differences in parental motivations (i.e., reasons underlying their decision to participate in the study), quality of activity practice at home (i.e., how closely home practice resembled the intervention activities), and the general quality of the child’s surrounding environment (i.e., access to physical equipment and social support). However, as the imbalance in parental education was not accompanied by differences in household income levels, or baseline levels of parental engagement, it is difficult to determine the true effect of this statistical imbalance. Nonetheless, it should be considered a limitation of our sample. Second, our sample was small and relatively homogenous; therefore, the generalizability of the results is limited. Furthermore, the power of our analyses is limited by the small sample; the analyses of the effect of the intervention on the subdomains of motor skills and parental engagement levels were substantially underpowered, and this may explain their non-significance.

## Conclusion

The results of this study show that participation in a direct instruction, community-based, parent-oriented movement and preliteracy program can significantly improve movement and preliteracy skill levels of preschool children with typical development. These novel results highlight the feasibility and importance of intervening during the preschool years and involving the parents throughout the intervention to maximize gains. Future research should begin to investigate the generalizability of the program in different community settings with larger more diverse samples.

## Ethics Statement

This study was carried out in accordance with the recommendations of Hamilton Integrated Research Ethics Board with written informed consent from all parents of participating children. All parents of participating children gave written informed consent in accordance with the Declaration of Helsinki.

## Author Contributions

CB designed the study, coordinated recruitment and data collection, designed the preliteracy component of the intervention and assisted with weekly implementation of the intervention, carried on the data analyses, and drafted the initial manuscript. EB assisted with study design, recruitment and data collection, design of the motor component of the intervention and weekly implementation of the intervention, and revised and approved the final manuscript as submitted. WC assisted with design of the intervention, selection and design of outcome measurements, and reviewed and approved the final manuscript as submitted. JC supervised the design and execution of all phases of the study and revised and approved the final manuscript as submitted. All the authors approved the final manuscript as submitted and agreed to be accountable for all aspects of the work.

## Conflict of Interest Statement

The authors declare that the research was conducted in the absence of any commercial or financial relationships that could be construed as a potential conflict of interest.

## Abbreviations

PWPA, preschool word and print awareness; PALS-PK, phonological awareness literacy screening-preschool; PDMS-2, peabody developmental motor scales-2nd edition; ANCOVA, analysis of covariance.
